# DDoS Flood and Destination Service Changing Sensor

**DOI:** 10.3390/s21061980

**Published:** 2021-03-11

**Authors:** Fu-Hau Hsu, Chia-Hao Lee, Chun-Yi Wang, Rui-Yi Hung, YungYu Zhuang

**Affiliations:** Department of Computer Science and Information Engineering, National Central University, No.300, Zhongda Rd., Zhongli District, Taoyuan City 320, Taiwan; hsufh@csie.ncu.edu.tw (F.-H.H.); 101582016@cc.ncu.edu.tw (C.-Y.W.); ian955246@gmail.com (R.-Y.H.); yungyu@acm.org (Y.Z.)

**Keywords:** DDoS attack, live migration, TCP three-way handshake, network security, loadable kernel module

## Abstract

In this paper, we aim to detect distributed denial of service (DDoS) attacks, and receive a notification of destination service, changing immediately, without the additional efforts of other modules. We designed a kernel-based mechanism to build a new Transmission Control Protocol/Internet Protocol (TCP/IP) connection smartly by the host while the users or clients not knowing the location of the next host. Moreover, we built a lightweight flooding attack detection mechanism in the user mode of an operating system. Given that reinstalling a modified operating system on each client is not realistic, we managed to replace the entry of the system call table with a customized sys_connect. An effective defense depends on fine detection and defensive procedures. In according with our experiments, this novel mechanism can detect flooding DDoS successfully, including SYN flood and ICMP flood. Furthermore, through cooperating with a specific low cost network architecture, the mechanism can help to defend DDoS attacks effectively.

## 1. Introduction

According to Kaspersky, there were approximately 250 distributed denial of service (DDoS) attacks every day in first quarter (Q1) 2019 [1]. DDoS attacks usually involve gigantic commercial losses worldwide. Business services online have naturally become prime targets. Given that services online are provided for general external users, an edge gateway is the channel of the internet service and external users. We must confirm that these critical places are stable and available. Thus, it is inevitable to set a solid detection mechanism on edge servers for security of their services. A DDoS attack usually arranges a huge number of internet bots, which can launch attacks on one specific target, such as an internet service or a network edge server. An attacker may start a DDoS attack from exploiting vulnerability of a specific system. For example, the Mirai botnet exploits the vulnerability of a default password [2]. If an attacker has compromised enough numbers of bots, the attacker can launch DDoS attacks by a command-and-control (C&C) system. We have a specific network security research environment that is based on the movement of the server to achieve the effect of protecting a service from a DDoS attack. In this paper, we call it the Method for Moving Virtual Machine (MVM), since a host that is under a DDoS attack cannot establish any new connection. While encountering a DDoS attack, those users who would like to establish new connections to the attacked server will also become participants in the attack, although they are not intentional. To overcome this shortcoming, we propose a new concept to detect flood attacks and establish a Transmission Control Protocol/Internet Protocol (TCP/IP) connection by the host that is under attack. First, we build this mechanism through Loadable Kernel Module (LKM) in order to replace the system call table entry with a customized sys_connect function. Moreover, this mechanism can transfer a request to establish a new connection to another proxy that is not under attack. When a proxy is under a strong DDoS attack, the proxy cannot tell which synchronize (SYN) packet is regular. Thus, we design a mechanism which responds to a SYN packet with a chosen probability for the proxy. If a SYN packet is chosen, the proxy responds to the sender with a SYN_ACK packet. If the proxy receives a corresponding acknowledgment (ACK) packet, the sender is an existing host. Next, we set a flood detection mechanism, which is lightweight, effective, and easy-to-use to detect DDoS attacks.

In subsequent sections, we introduce the Client Connection Handler and discuss the continuous connection when the destination of a service changes; moreover, we review the entire scenario and describe each component in detail, including a practical environment where this sensing system can be applied. In Section 2, we conduct a thorough literature review. In Section 3, we introduce the background of our system. In Section 4, we provide an overview of our system. In Section 5, we describe the mechanism that can defend against DDoS attacks in detail. We show the efficacy of our system in Section 6 and present a discussion in Section 7, before concluding.

## 2. Related Work

This methodology can be used in a non-virtual machine environment and a virtual machine environment (we used both in experiments). In regards to virtual machine technology, two research studies on the prototype are the VM Turntable demonstrator by Travostino et al. in 2006 [3] and by Bradford et al. in 2007 [4]. VM Turntable demonstrates a prototype of live migrating for virtual machines on a wide area network (WAN). VM Traffic Control is used to determine the target and control the process of live migration. The path of live migration is based on a special lightpath. In addition, the source machine, destination machine, and the clients can stay connected under a same subnet by the mechanism of an IP tunnel. Travostino et al. proved the possibility of live migration. However, it must modify all of the switches on the path to build a lightpath, and the control system is too vulnerable. It is not appropriate to deploy this prototype in the real world. In addition, to save costs, and for the ease of management and maintenance, many users prefer virtual machines to physical hosts. Infrastructure as a service (IaaS) has gradually replaced traditional hosting services, acting as a new alternative for many enterprises and individuals to set up or to deploy their servers. Similar to the Amazon Elastic Compute Cloud (Amazon EC2) [5] and Google Cloud Platform (GCP) [6], IaaS has experienced explosive growth, becoming popular in recent years. On the other hand, in cyber architecture, the solutions for current DDoS attack problems adopted by industries are often through content distribution networks (CDNs), flow cleaning, or even worse, by just using a firewall. Famous corporations that focus on mitigating DDoS attacks with CDN are Cloudflare [7], Akamai [8], and Alibaba Cloud [9]. However, to corporations’ disappointment, it is just “mitigation” and not a comprehensive defense. In addition, some methods that are similar to network traffic cleaning at a lower level can be expensive and not as effective. Generally speaking, they cannot really resolve the problem effectively. CDN or load-balance needs to deploy a lot of resources. In regards to flow cleaning, it costs much higher.

Because our research highlights the concept of dynamic defense to protect services from DDoS attacks, we investigate and discuss relevant research on this topic. First, when it comes to DDoS traffic detection, three DDoS detection papers are worth mentioning. One is by Hussain et al. [10], who claimed to analyze the headers of the packets in the traffic in a simple way; they summarized their “spectrum” with some mathematical formulas (it will be effective to filter out the malicious traffic). However, according to our research, in practice, this way has not been appropriate of late due to heavier traffic; moreover, this approach seems too slow to act immediately and useless at protecting services against DDoS attacks. Another (earlier) paper is by Carl et al. [11]. This paper described many kinds of detection methods, but all of them are, likely, impractical. The authors also noticed that none of them completely solved the detection problem. The other paper is by Dainotti et al. [12]; this paper claimed that the “adaptive threshold” and “cumulative sum” with the continuous wavelet transform provides good results on the detection of DDoS attacks. However, we suggest that it makes it too complicated, and we can get very good and efficient results with our concise method, nowadays, in the practical network environment. Additionally, we refer to mechanisms related to DDoS defense, which have been proposed academically by researchers in recent years. In 2007, Bradford et al. [4] proposed a way to keep the connection for live migration in a WAN. After live migration, the source machine is responsible for forwarding the packets of the old connection, and the virtual machine uses the destination machine to update dynamic Domain Name System (DNS) to receive new connections on another network interface. This method is suitable for the service, which has a short connection time, such as HTTP, to let the source machine go out as soon as possible.

A moving target DDoS defense mechanism (MOTAG) [13] deploys a group of dynamic proxy nodes that relay traffic between servers and authenticated clients, providing a moving target defense mechanism that mitigates internet service DDoS attacks. Each client can only know the IP address of the proxy node, which is randomly assigned. Moreover, its authentication server is responsible for important tasks, such as the authentication of all its clients, the proxy assignation, etc. However, if just one attacker launches a significant DDoS attack to this authentication server, this defense mechanism will crash easily. Thus, it is a considerable disadvantage of this design, and the architecture is still fragile under any DDoS attack. “Catch Me If You Can” [14] is just like a modified version of MOTAG, which is also a moving target mechanism. It is a cloud-enabled moving target defense mechanism, and its deployment is across multiple cloud computing domains. The main idea of this work is to use a DNS server and a coordination server, with cloud content servers of a large load balance mechanism. Nevertheless, this mechanism has the same disadvantage of concept: if the DNS server (or the coordination server) is under attack, this defense structure will be vulnerable and unbearable to an expressive DDoS attack. Moreover, this mechanism will need substantial resources for construction at first. Finally, its coordination server (which is the role of a central controller) may have a heavy load, and may have efficiency issues, in practice.

This kind of attack brings considerable damage to businesses; however, there are still new mechanisms for mitigation. Gaurav Somani et al. proposed a DDoS attack mitigation method by lowering the “Resource Utilization Factor” to a minimal value and discussing the algorithm [15]. Sunny Behal et al. proposed an Internet service provider (ISP) level distributed and flexible defense system [16]. Patil, R. et al. designed a Protocol Specific Multi-Threaded Network Intrusion Detection System (PM-NIDS), where the incoming packets are queued, extracted, and classified [17]. Chenxu Wang et al. proposed a defense system of detecting and mitigating application layer DDoS attacks, mainly by filtering mechanisms, including a whitelist and a blacklist [18]. Lei Cheng et al. proposed a moving target defense technique based on self-adaptive mutation, consisting of a network threat awareness mechanism based on Sibson entropy and a mutation strategy algorithm [19].

The above defense solutions of DDoS attacks are usually complicated and cannot really solve the problems—most of them are just for mitigation. The MOTAG mitigation series requires another specific server, at least to coordinate its processing. Furthermore, it requires a lot of resources. The mechanism in this paper is “decouple”, and each proxy server is only activated or built when needed. There is no need for huge hardware resources to support the system. Our system is novel and the design details are not the same as the previous work.

## 3. Background

### 3.1. Context

The context of changing another destination may be triggered when a service is under attack by a DDoS attack. Hereafter, we will call this mechanism the Migration Sensor (MS).

### 3.2. DDoS Attacks

The protection of network services on the internet needs to be discussed. However, concerning DDoS attacks, for decades, all solutions had no perfect defense mechanism. To put it bluntly, there is no effective solution to defend against DDoS attacks. Thus, it is still the biggest challenge to business services on the internet. Reports show that DDoS attacks have become larger and more powerful in recent years. The most recent and largest DDoS attack, according to Impera, occurred in 2019. This attack peaked at 580 million packets per second (PPS), which was an even larger PPS attack on its client, surpassing the January record of 500 million PPS [20]. In comparison, another famous DDoS attack was launched in GitHub in 2018, peaking at 129.6 million PPS.

On 28 February, 2018, GitHub, a popular online code management service utilized by millions of developers, was hit by a DDoS attack at 1.35 terabits per second. After approximately 8 minutes, GitHub called Akamai, the company tasked with DDoS attack mitigation for GitHub. This was the largest DDoS attack ever recorded at the time. It was a memcached DDoS attack: the attackers leveraged the amplification effect of a database caching system known as “memcached”. By flooding memcached servers with spoofed requests, the attacks were amplified by a magnitude of about 50,000 times [21].

Another famous (and large) DDoS attack involved the Internet of Things (IoT) in October 2016. This DDoS attack was caused by Mirai, creating a botnet from compromised IoT devices, such as cameras, smart TVs, and monitors. The flow of this event was directed at Dyn (a major DNS provider) and resulted in devastating consequences for many major sites, including Netflix, PayPal, Visa, Amazon, and GitHub. With Mirai, attackers can program the compromised devices and send requests to a single victim. After that, in February 2020, Amazon Web Services (AWS) reported that they defended against a 2.3 terabits per second DDoS attack [22]. This trend will only continue to increase.

According to research from Corero Network Security, DDoS attacks can cost enterprises USD $50,000 (£35,000) per attack. The survey showed that individual DDoS attacks can cost organizations up to $50,000 in terms of lost business, the cost of mitigating attacks, and lost productivity. Moreover, 69% indicated that their organization experienced between 20 and 50 DDoS attack attempts each month, which is equal to, on average, one attack per day. DDoS attacks not only immediately—and considerably—impact the revenue of enterprises (by causing loss of earnings), these attacks also damage the trust and confidence of customers [23,24,25].

### 3.3. Flood Attack

There are other categories of DDoS/DoS attacks, such as a slow DoS attack, but a flood attack is the most common type of DDoS attack. A flood attack relies on a large number of packets to paralyze the server by exhausting the bandwidth or by continuously consuming server resources. The type of flood attack has an obvious feature, such as sending a large number of packets in a short time, and it is common (meanwhile, a slow attack achieves the same goal in other tricks, including delaying the delivery of some content). Thus, we will use the flood attack as a representative type of a DDoS attack.

### 3.4. Linux System Call

**Figure 1:**
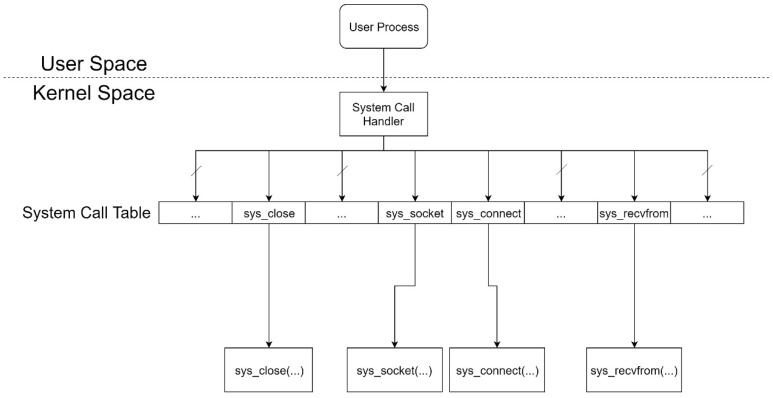
Original structure of a Linux system call.

As seen in Figure 1, the original Linux system call process is called by a user process, and then the execution flow switches to kernel mode. After a system call handler completes system call preparation, the execution flow jumps to a system call block, in accordance with a corresponding system call table entry.

## 4. System

### 4.1. Method for Moving Virtual Machine (MVM)

The purpose of this work is to defend against network based DDoS attacks. For the system, it contains a client agent, proxies, and a server. The client agent is responsible for handle connection migrations on the client side. Proxies forward packets, detect possible DDoS attacks, and migrate connections. The server is a virtual machine that provides network services. In a system overview, we deploy a client, two proxies, and a server. In the following sections, we call the proxy given to clients as Proxy 1, which will be attacked; the other one is Proxy 2, which will receive an immediate notification from Proxy 1.

**Figure 2:**
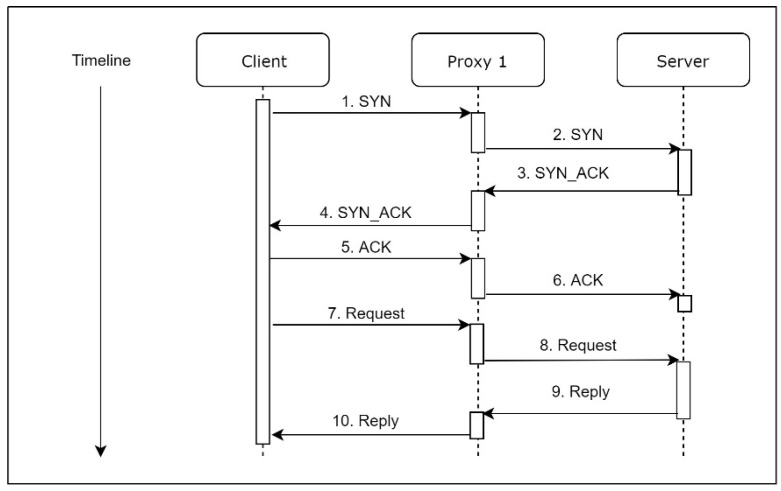
Moving Virtual Machine (MVM) workflow under normal conditions.

In normal situations, which means there is no DDoS attack, Proxy 1 works just like a simple proxy server, as shown in Figure 2.

**Figure 3:**
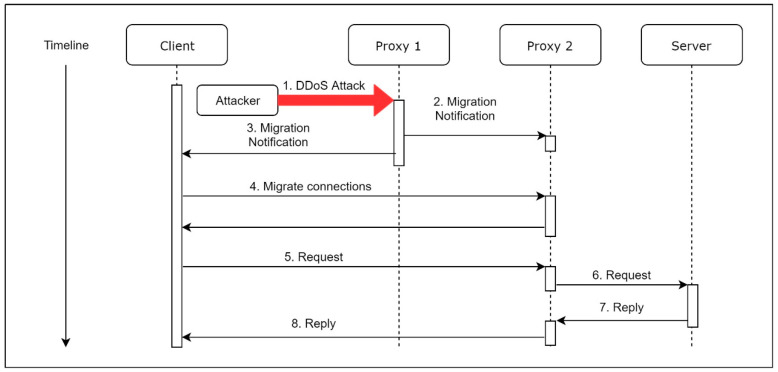
MVM workflow under distributed denial of service (DDoS) attack.

When Proxy 1 is under a DDoS attack, as shown in Figure 3, Proxy 1 will send two notifications. The first notification, which contains the current user connections’ information, is sent to the Proxy 2, so Proxy 2 can setup its whitelist, depending on the notification. The second notification contains the IP address of Proxy 2, which is sent to the connected clients. After a client receives the notification, the client will start a migration process. Once the migration process is complete, the client communicates with the server through Proxy 2. Besides, the IP address of Proxy 2 remains unknown for all clients until the migration starts. Furthermore, the dispatched Proxy 2 may be more than one destination, which means there are different machines of Proxy 2 for the connected clients so that the adversary can never launch a successful DDoS attack to all of the proxies at the same time.

### 4.2. Sensing System

This paper introduces a simple Ethernet packets flow detection mechanism for the detection. It monitors the number of packets at the frontier of a server. This flood-detection module will calculate the volume of the packets of the Ethernet in every second. We need to set a threshold to the module, manually, on each of the various conditions. If the volume in a second exceeds the abnormal threshold, the flood-detection module will judge it as a suspected DDoS attack. According to our experiments, it can detect flooding DDoS attacks successfully, including SYN flood attacks and ICMP flood attacks. On the other hand, in this work, we call the process of establishing a new connection by Proxy 1, which is under DDoS attacks, as “transfer”. The main difference between “transfer” and “migrate” is whether a connection has been established; “migrate” requires an established connection, but “transfer” does not as yet.

**Figure 4:**
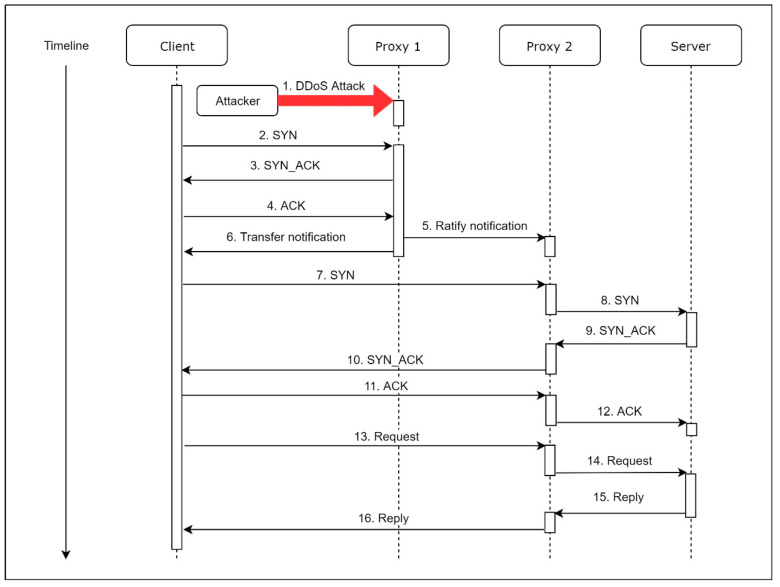
Execution flow of Migration Sensor (MS) under DDoS attacks.

For MS, the most different functionality from the MVM is that the MS can transfer a request for establishing a new connection to the proxy that has not been under attack. As shown in Figure 4, when a proxy is under a strong attack, the proxy is unable to determine which SYN packet is a regular one. The proxy responds to a SYN packet with a chosen probability. The proxy responds to the SYN packet of the sender with a SYN_ACK packet if a SYN packet is chosen. If the proxy receives a corresponding ACK packet, this indicates that the sender is a real host.

Since the sender is verified as an existing host, we send a transfer notification, which contains the IP address of Proxy 2, to the sender. Besides, we send a ratify notification, which contains the information about the client, to Proxy 2, so that we can update the firewall rules on Proxy 2.

### 4.3. System Overview

The MS system can be divided into three parts: clients, proxies, and servers. A client has a Connection Handler. A proxy mainly contains a DDoS Detector, a Packet Handler, a SYN Checker, and an Informer. The server provides network service, such as Secure Shell (SSH), HTTP/HTTPS service, and so on.

**Figure 5:**
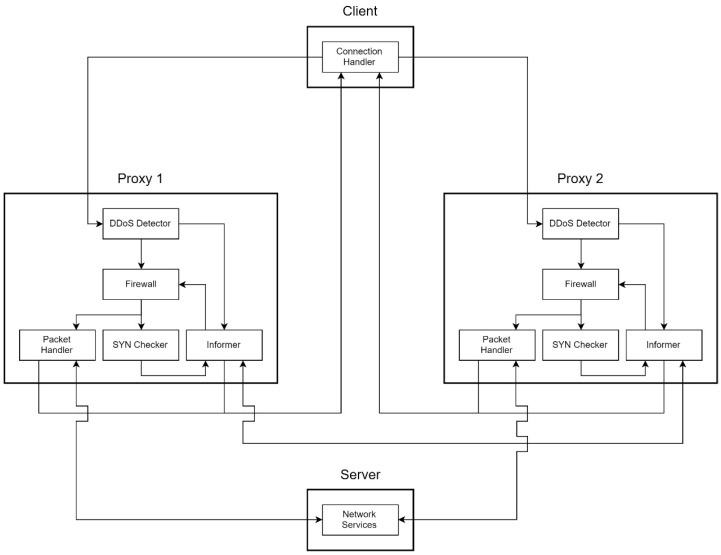
System overview.

The simplest structure contains a client, two proxies, and a server, as shown in Figure 5. The most important sensing components of MS are Connection Handler and DDoS Detector.

#### 4.3.1. Connection Handler

Connection Handler is in charge of establishing a new connection. The Handler makes a three-way handshake first, then tries to receive a transfer notification until timeout. If the destination host sends a transfer notification back, which contains the secret word and the IP address of another proxy, the handler rewrites the destination IP of the struct sockaddr with the IP written inside the transfer notification. After rewriting, the Connection Handler makes another three-way handshake to establish a new connection. For easy identification, hereafter, Client Connection Handler will be called CCH.

#### 4.3.2. DDoS Detector

DDoS Detector is a main module of MS, which is to sense the number of packets at the frontier of the network architecture, which is in charge of detecting flood traffic. It is a lightweight detection mechanism against flooding attacks. For friendly use, we develop it simply in the user mode of an operating system. A network administrator can set the configurations easily and start immediate protection. Once it detects unusual traffic, the detector will send a signal to Informer. We will discuss the functions of Informer later.

#### 4.3.3. Packet Handler

Packet Handler is in charge of forwarding packets. Moreover, Packet Handler is in response to the process connection migrating progress, after a DDoS attack detected by the DDoS Detector.

#### 4.3.4. SYN Checker

After DDoS Detector detects DDoS attacks, Informer sets the firewall, and switches the callback from Packet Handler to SYN Checker. SYN Checker selects a SYN packet to respond with chosen probability. That is, it does not respond to all of the SYN packets. If the client is a normal client rather than a forged source produced by an attacker, it will respond ACK after it got the SYN_ACK. Since the SYN Checker receives the correspond ACK, it can ratify the connection, then call Informer to send the transfer notification to the client and the ratify notification to the next proxy (Proxy 2) to maintain the original connection of the service from previous proxy (Proxy 1).

#### 4.3.5. Informer

Informer tries to send notifications and manages the firewall rules. When the DDoS Detector detects DDoS attacks, the DDoS Detector sends a signal to Informer. After Informer receive the signal, it sends migration notifications to the other proxies and clients that establish connections.

## 5. Implementation

For establishing a new connection through our proxy, which is under a DDoS attack, the mechanism of MS covers the transfer progress. We suppose that MS is under a strong attack and SYN Checker can accept the connection requests from all clients of this system. At first, Proxy 1 detects the attack, and Informer sets up the firewall. A client would like to establish a new connection through Proxy 1 at this point. If SYN Checker collects this SYN packet, with chosen probability, Informer sends the corresponding SYN_ACK packet to the client and sets the firewall rule to allow the possible corresponding ACK packet.

Next, it sends an ACK packet to Proxy 1 after the client receives the SYN_ACK packet. Because the client has been verified as a regular client by SYN Checker, Informer sends an approval notice to Proxy 2 and a transfer notification to the client. The approval notice contains the information of the client, which will establish a new connection to Proxy 2, and then Proxy 2 can set its inner firewall policy to allow this client to communicate; the transfer notification carries the IP address of Proxy 2 so that the client can establish another connection with it, which is under a normal situation.

CCH sends the SYN packet with a new destination. The packet will pass through the Detector and the firewall on Proxy 2, and then Packet Handler sends it to the original server. Finally, after the server sends the SYN_ACK back to Proxy 2, Packet Handler will forward it to the client. Once the transfer process finishes, the new proxy (Proxy 2) operates as a new frontend proxy, while the previous proxy (Proxy 1) is set as a temporary suspension state.

### 5.1. Design

**Figure 6:**
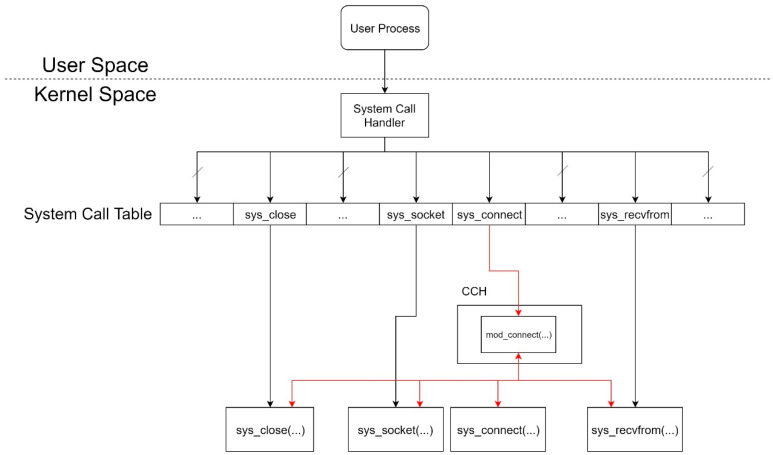
Workflow of Client Connection Handler (CCH).

The important point of the Migration Sensor (MS) system mainly concerns the design of Detector and CCH. Thus we focus on describing these two significant components. When there is a malicious DDoS attack, Detector will distinguish it and trigger the next procedure to notify the migration. Detector will check the volume of flood packets with the threshold set by the network administrator earlier. Then, CCH takes over the next processing step. As shown in Figure 6, we insert a table entry with the address of our customized sys_connect, which is named mod_connect. Thus, we can create a socket and receive transfer notifications just in the kernel space.

### 5.2. Conditions of CCH Workflow

**Figure 7:**
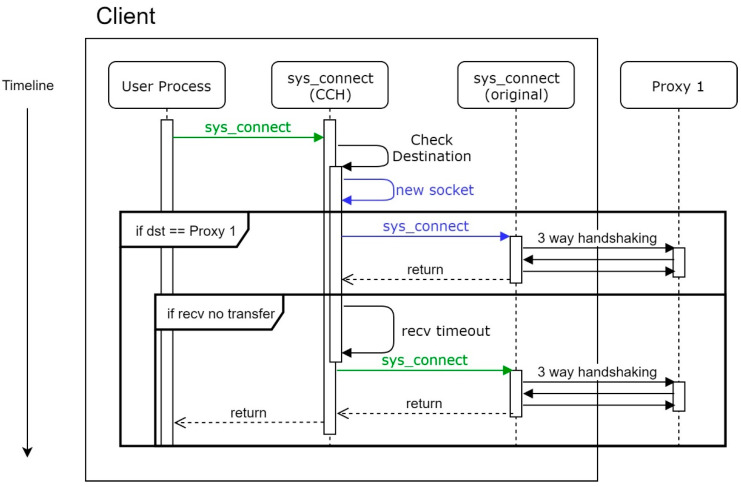
Workflow of CCH under a normal situation.

Under a normal situation, the MS system will conduct three-way handshaking twice, as Figure 7. The first three-way handshake is to check if the proxy intends to conduct the transfer process. Supposing Proxy 1 is unprotected, a client will not receive any transfer notification, which is caused by a receive timeout. Moreover, every time the sys_connect is called, the CCH will conduct this process. Otherwise, the client cannot know the state of the proxy until the proxy sends a notification.

**Figure 8:**
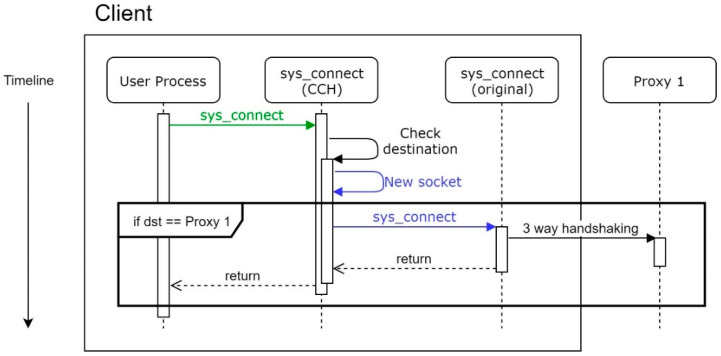
Workflow of CCH when no response.

For some reason, a client may not get the response from the proxy, as Figure 8. For instance, the proxy is under a strong DDoS attack, and SYN Checker on the proxy is forced to omit this SYN packet. We will not retry in CCH in this case, but only let the user process make a decision.

**Figure 9:**
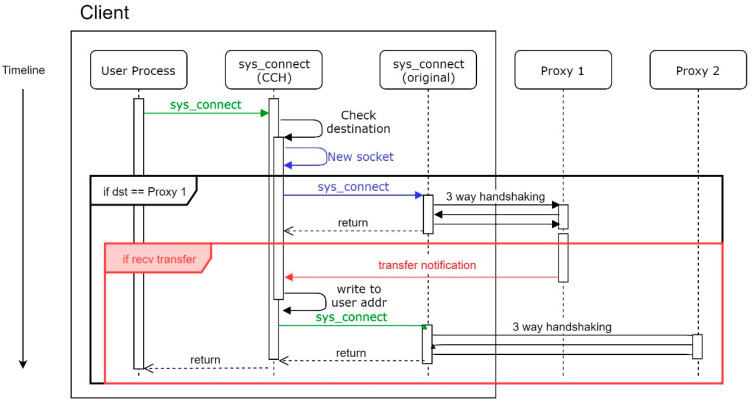
Workflow of CCH when the Proxy 1 is under attack.

On the other hand, while the MS system is enabled and Proxy 1 encounters a DDoS attack, SYN Checker of Proxy 1 that is under attack will respond to SYN packets with a chosen probability as mentioned in Section 4.3.4. It can check the availability of the source by a three-way handshake before SYN Checker sends a transfer notification. Proxy 1 sends a transfer notification to the client after the client responds with a corresponding ACK packet, which means the client is a real user rather than a forged source. Once CCH receives this notification, CCH rewrites the destination with the IP of another proxy, which is recorded in the transfer notification. The workflow is depicted as shown in Figure 9.

As a result, CCH makes a three-way handshake with the new destination and establishes a new connection.

## 6. Evaluation

In this section, we evaluate the performance overhead under selected common situations. Each experiment includes evaluated benchmarks. The micro benchmark measures the time consumption of establishing a new connection for 1000 times and gets the average value. The macro benchmark measures the time consumption, also repeated for 1000 times. Nevertheless, the macro benchmark is closer to the real world. We measure the time consumption of downloading a 760 kilobytes picture from a server. In order to ensure that the download process works properly, we compare the MD5 value of each image.

### 6.1. Experimental Specification

The system specifications of the client and the two proxies are shown in Table 1.

### 6.2. Testing Existing Functionality

When it comes to detecting DDoS attacks, Detector of MS can always detect the flood of DDoS attacks, with an appropriate threshold. In conclusion, the success rate is 100% when attackers launch flood DDoS attacks. The other experiment focuses on functionality. Though we have modified the behavior of sys_connect, the original functionality should still work (as the same as before). In this case, we took the website of the White House as a destination, measured the time consumption of a three-way handshake, and then downloaded an image by HTTP protocol. As mentioned, each test was repeated 1000 times. Testing existing functionality also means that MVM does not perform together on this experimental architecture, which is a pure functional test for the MS system.

**Figure 10:**
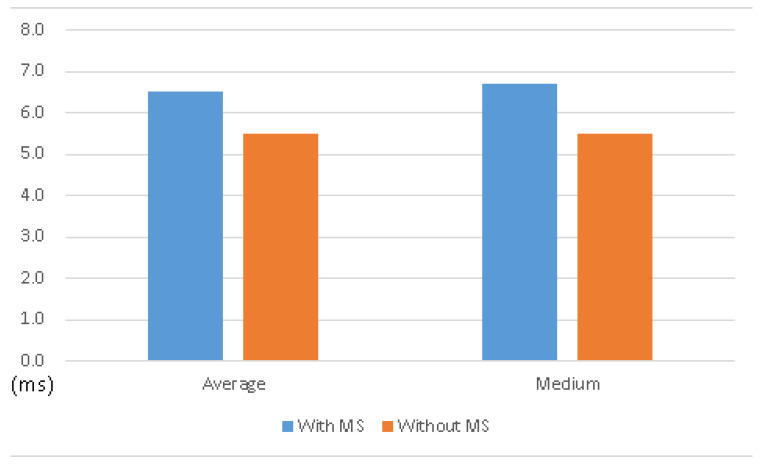
Average time and medium time of testing existing functionality—new connection.

**Figure 11:**
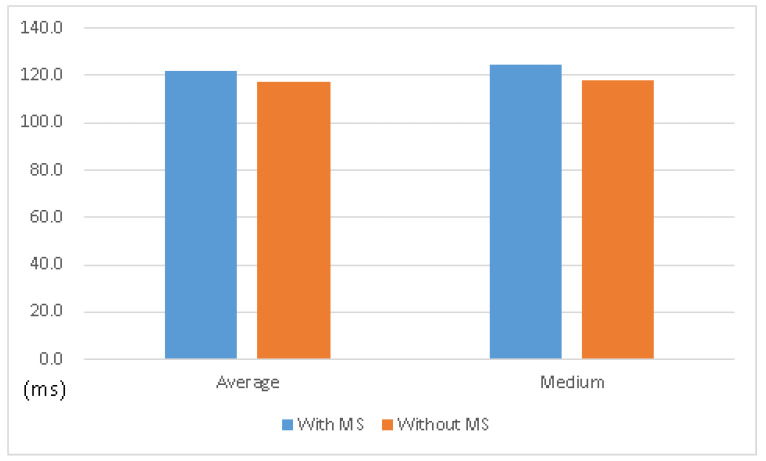
Average time and medium time of testing existing functionality—download data.

In comparison with the operating system without MS, the one with MS comes with almost the same performance, as shown in Table 2, and Figure 10 and Figure 11. It shows that the MS system can keep the original functionality with acceptable overhead.

### 6.3. Connecting to Protected Server under a Normal Situation

We need to consider when MS will be used in practice. In order to defend against DDoS attacks and protect a server, the MS system must work together with MVM to complete the defense procedures. This experiment focuses on the overhead of a client in the MS system under a normal situation; that is, it is combined with MVM and is not under attack. In the experiment, we assume that Proxy 1 is protected. In addition, to consider in the next experiment, we simulate a DDoS attack on Proxy 1, we deploy the whole system in an isolated local network. As a proof of concept, the IP address of Proxy 1 was hardcoded in current CCH in the test. Nonetheless, the IP address of Proxy 2 can still be changed or selected at any time.

**Figure 12:**
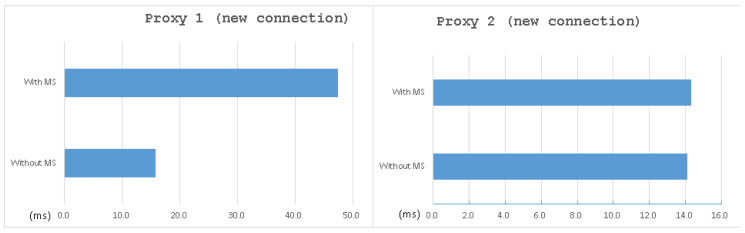
Average time of Proxy 1 and Proxy 2 when defending against DDoS attacks—new connection.

**Figure 13:**
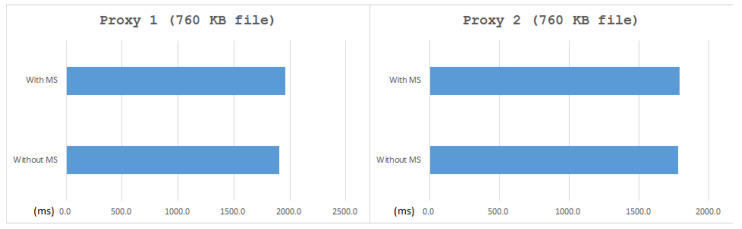
Average time of Proxy 1 and Proxy 2 when defending against DDoS attacks—downloading image.

Moreover, since the HTTP functionality of the MVM system is a work in progress, we download the image by SSH in this test. As shown in Table 3, the overhead of establishing a new connection after installed MS came to almost 200%. However, as shown in Table 4, the overhead of downloading an image only came to 2.8%. We also give some figures for immediate understanding of the result, as see in Figure 12 and Figure 13. It shows that, except for the overhead of the new connection of Proxy 1, the overhead of the whole system (including MVM) was little, which is always less than 3% on average.

The reason why there is a dramatic difference between the two benchmark is that once the three-way handshake finishes, the sys_connect will not be called again until a new connection is required. In other words, there are many data transmissions after a three-way handshake for a real scenario, causing the client to spend more time on data transmissions. As a result, MS comes with a significant overhead in establishing a new connection, but it is not a big issue in a functional case.

### 6.4. Connecting to Protected Server under Simulate DDoS Attacks

In this section, we assume that Proxy 1 is under a DDoS attack. To simulate the SYN flooding attack, we took hping3 as an attack launcher. The attacker host sent SYN packets with a random source every 1000 microseconds.

As seen in Table 5, the result shows that it is possible to establish a new connection through the proxy being attacked. It also shows that the successful rate can be increased by retries, which were made by the user process. Unfortunately, this novel mechanism is mostly different from the previous related work, so there is no way to do similar functional comparisons.

## 7. Discussion

In this work, we use a firewall as an extra traffic filter, which reduces the traffic load so that SYN Checker can deal with it. The reason why we designed this additional component is that we would like to adapt an existing system MVM to our solution, but the existing MVM implementation came with serious performance issues during our pressure tests. As a stable solution, we deployed a firewall to deal with performance issues. On the other hand, SYN Checker can also be a traffic filter, which picks up a SYN packet randomly to respond and drop others. In the future, we have to improve the performance of the components on the proxy to make SYN Checker work with an attack traffic. We search the system call table from an exported symbol sys_close to the other exported symbol loops_per_jiffy, and we add the size of the pointer with the accumulator, which is a register in a computer’s central processing unit (CPU). It works properly on Ubuntu 18.04 with the kernel Linux Ubuntu 4.15.0-29-generic, but this LKM usually cannot install on Ubuntu 18.04 with the updated kernel Linux Ubuntu 4.15.0-54-generic.

Essentially, if we combine MS system with MVM, the entire system will be superior to previous defense solutions, as it can transfer the service to another public IP address, in this way, at any time, and also notify both old users and new users, at a low cost. Previous work lacks this mechanism and cannot move the server to another public IP address. Moreover, solutions nowadays usually need a lot of resources. Even more, their system usually depends on a few more vulnerable servers that handle their defense mechanisms. Once these servers are crashed by malicious behaviors, including a DDoS attack, the whole defensive mechanism still shuts down.

## 8. Conclusions

In this paper, we propose a kernel module based mechanism to establish a new TCP/IP connection by the host that is under attack (the clients did not know the location of the host at first). In addition, it combines a module to monitor the number of packets at the frontier of its network architecture to detect DDoS attacks. This novel mechanism is called Migration Sensor (MS). When a flood attack occurs, MS will trigger a series of procedures to set up another location and change the service destination to the new location. Although it takes almost 200% overhead on three-way handshaking, the overall overhead only takes 2.8%, which is so low that it can be seen as an error. The simulation also shows that MS can improve the availability of the server, which is available for established connections under the Method for Moving Virtual Machine (MVM). Thus, we consider that MS is a successful proof of our design.

When it comes to the function of DDoS attack detection, we consider some previous work makes it too complicated and we propose a concise lightweight DDoS Detector module, which has very efficient results in the general network environment. We designed a novel component, Client Connection Handler (CCH), to make another three-way handshake to establish a new connection for the destination transferring. Besides CCH, we also discuss a whole practice scenario where this sensing system can be applied, and depict each component. If the MS mechanism runs in the MVM environment, the entire system can transfer the service to another public IP address at any time and also notify both old users and new users without service disconnection. Previous work lacked this novel mechanism and usually required more resources than our system, were often complicated, and either had huge hardware resources or a few fragile servers to support the system.

## Figures and Tables

**Table 1:** System specification.

	Client	Proxy 1	Proxy 2
Operating system	Ubuntu 18.04 AMD64 (Desktop)	Ubuntu 18.04 AMD64 (Server)	Ubuntu 18.04 AMD64 (Server)
CPU	Intel Core 2 Quad Q9400 @ 2.66 GHz	Intel Core i7-2600 @ 3.40 GHz	Intel Core i5-4460 @ 3.20 GHz
RAM	DDR2 4 G	DDR3 16 G	DDR3 16 G
Type of system disk	HDD	HDD	HDD

**Table 2:** Testing existing functionality.

	Without MS		With MS	
Test Case	New Connection	Download Data	New Connection	Download Data
Success rate	100%	100%	100%	100%
Average (ms)	6.5	121.9	6.7	124.3
Medium (ms)	5.5	117.3	5.5	118.1
Standard Deviation	5.5	20.7	5.8	26.2
Overhead of MS			3.1%	1.9%

**Table 3:** Establishing a new connection under a normal situation.

	Without MS		With MS	
Destination	Proxy 1	Proxy 2	Proxy 1	Proxy 2
Success rate	100%	100%	100%	100%
Average (ms)	15.8	14.1	47.4	14.3
Medium (ms)	14.5	13.1	45.6	13.1
Standard Deviation	7.2	6.6	9.9	8.8
Overhead			199.9%	1.4%

**Table 4:** Downloading a 760 KB image under normal situation.

	Without MS		With MS	
Destination	Proxy 1	Proxy 2	Proxy 1	Proxy 2
Success rate	100%	100%	100%	100%
Average (ms)	1902.3	1776.2	1956.0	1789.0
Medium (ms)	1899.3	1763.2	1947.9	1760.7
Standard Deviation	35.1	87.1	45.3	56.1
Overhead			2.89%	0.7%

**Table 5:** Successful rate of three-way handshaking and downloading a 760 KB image.

Accept Probability	1	0.75	0.5	0.25
Three-way handshaking	2.4%	2.2%	1.3%	0.3%
Download an image	3.7%	3.1%	2.1%	0.9%

## Data Availability

The data presented in this study are available on request from the corresponding author.
